# Regadenoson Stress Testing: A Comprehensive Review With a Focused Update

**DOI:** 10.7759/cureus.12940

**Published:** 2021-01-27

**Authors:** Karim O Elkholy, Omar Hegazy, Adeniyi Okunade, Suat Aktas, Temitope Ajibawo

**Affiliations:** 1 Internal Medicine, Brookdale University Hospital Medical Center, New York, USA; 2 Internal Medicine, Mercy Hospital, Chicago, USA

**Keywords:** myocardial perfusion imaging, regadenoson, cardiac stress test, lexiscan, coronary stenosis, adenosine, socio-economic factors, pharmacological stress agents, nuclear stress test, non obstructive coronary artery disease

## Abstract

Regadenoson is a pharmacological stress agent that has been widely used since its approval by the Food and Drug Administration (FDA) in 2008. For many years, dipyridamole and adenosine, which are non-selective adenosine receptor agonists, were more popular. However, these agents are less preferred now due to their undesirable adverse effects as compared to regadenoson. In the ADVANCE (ADenoscan Versus regAdenosoN Comparative Evaluation) phase 3 clinical trial, regadenoson demonstrated non-inferiority to adenosine for detecting reversible myocardial ischemia. This review summarizes the clinical utilities of regadenoson as the most widely used pharmacological stress agent. Moreover, the use of regadenoson has been documented in specific patient populations. Although regadenoson has established safety and efficacy in most patients with chronic diseases, there are equivocal results in the literature for other chronic diseases. It is warranted to highlight that the use of regadenoson has not been studied in patients of low socioeconomic class; it is a condition that carries a significant burden on the cardiovascular system.

## Introduction and background

The main idea behind stress testing is to assess the extent and adequacy of the ability of the coronary circulation to augment flow, reflecting coronary flow reserve (CFR). Ischemic symptoms start when there is either abnormality in coronary supply or demand imbalance. Myocardial stiffness follows, inducing wall motion abnormalities. Resultant electrocardiographic ST-segment changes occur, and chest pain develops. Stress testing induced hypoperfusion, which diagnoses coronary artery disease (CAD) in the setting of flow-limiting coronary stenosis. Cardiac stress is achieved either by exercise or pharmacologically. Exercise affects CFR indirectly through increasing the heart rate primarily and thus increasing flow demand, which, in turn, results in increased flow. The endpoint of interest is wall motion abnormality, seen as ST-depression on electrocardiogram (ECG) [1]. Exercise stress testing is used in symptomatic intermediate-risk patients who can exercise and who have interpretable electrocardiography results. Risk is determined by the American College of Cardiology Foundation/American Heart Association’s (ACCF/AHA) guidelines for stable ischemic heart disease or the Diamond and Forrester score to assess the pretest probability of coronary artery disease [2]. Testing can be performed with or without myocardial perfusion imaging. Imaging such as single-photon emission computed tomography (SPECT) and positron emission tomography (PET) is usually used when there is a baseline abnormality in resting electrocardiography that would make the interpretation of results difficult if the patient has symptoms at rest or if anatomic cardiac features require evaluation [3]. Notably, its sensitivity is directly related to the ability to augment demand, thus exercise must be adequate for the appropriate evaluation, which ultimately imposes possible limitations on its use. An adequate diagnostic exercise stress test requires reaching 85% of the maximal predicted heart rate [3]. In patients who cannot fulfill this target due to abnormalities involving the respiratory system or having ongoing systemic problems limiting their mobility, or when baseline EKG is abnormal such as with left ventricular hypertrophy (LVH), left bundle branch block (LBBB), paced rhythm, Wolff Parkinson White (WPW) syndrome, or greater than 1 mm ST-segment depression, pharmacological stress testing is considered [4]. Similar to exercise, dobutamine, an inotropic direct agonist of mainly B1 receptors, affects CFR indirectly through increasing flow demand, affecting wall motion and resulting in ST-depression. It can replace exercise stress testing in patients who cannot exercise. Adenosine, dipyridamole, and regadenoson are coronary vasodilators. They exert their effect on CFR directly by increasing the coronary flow. In contrast to exercise and dobutamine, these agents induce perfusion heterogeneity in stenosed segments rather than inducing flow demand and subsequent ischemia. As a result, these agents need to be coupled with myocardial perfusion imaging [3]. They act through interacting with subtype 2a of the adenosine receptor (A2a). Adenosine is a naturally occurring, non-selective agonist of all subtypes of adenosine receptors. Adenosine deaminase is the enzyme responsible for its degradation. It is inhibited by dipyridamole, thus increasing the intrinsic adenosine concentration. This non-selectivity produces multiple undesirable side effects, notably, bronchoconstriction via adenosine receptor subtype 2b (A2b) stimulation, and bradycardia and atrioventricular block via adenosine receptor subtype 1 (A1) stimulation. In contrast, regadenoson is a selective A2a receptor agonist with a safer and better tolerability profile. Additionally, regadenoson is administered as a single bolus, weight-unadjusted dose, unlike the weight-adjusted infusion dose of adenosine and dipyridamole [5-7]. This ease of administration and the reproducible, comparable efficacy to adenosine with fewer side effects made regadenoson the most widely used pharmacological agent for stress testing in the United States [8].

## Review

**Figure 1:**
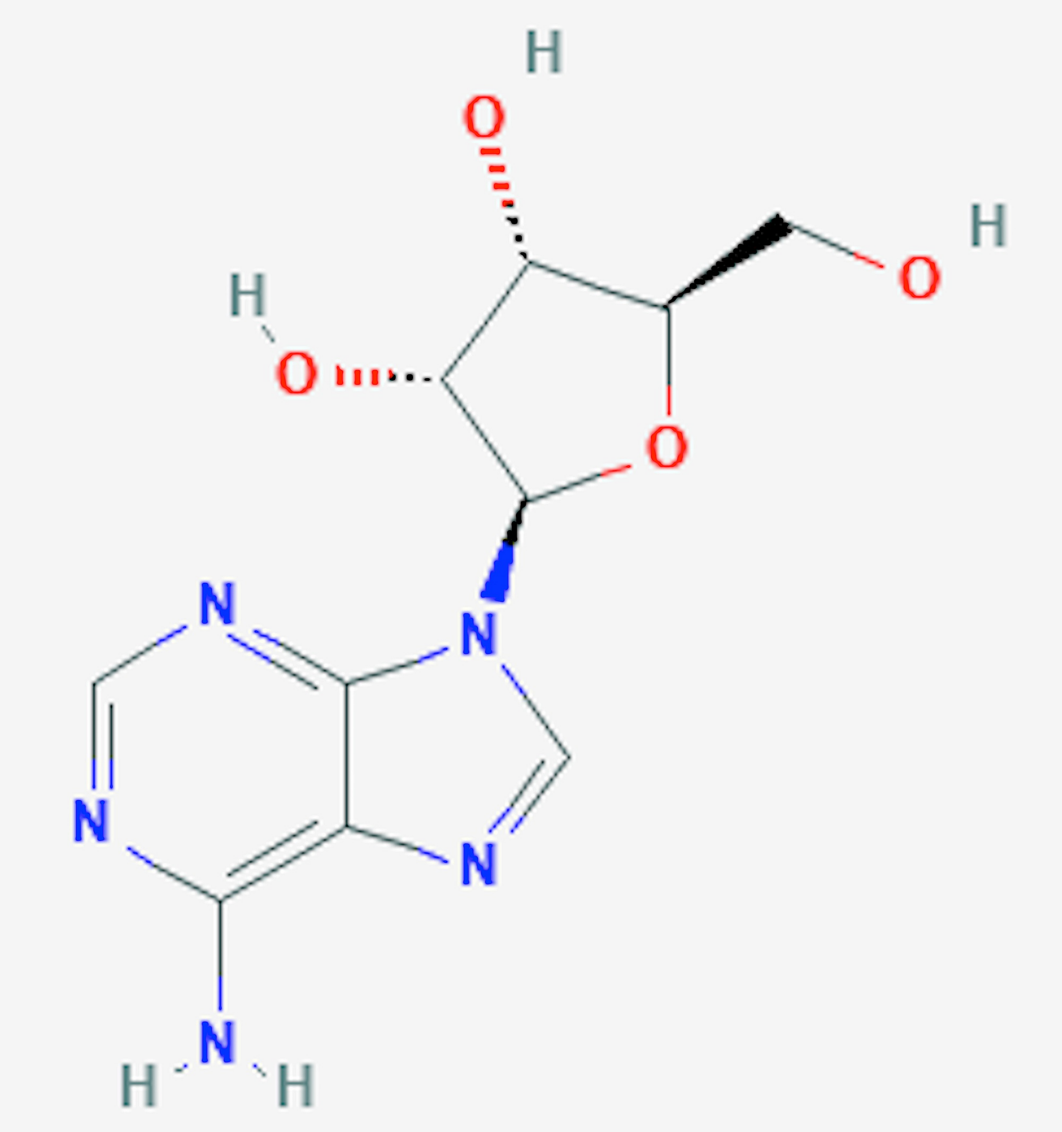
The chemical structure of adenosine National Center for Biotechnology Information (2020). PubChem Compound Summary for CID 60961, Adenosine. Retrieved October 13, 2020, from https://pubchem.ncbi.nlm.nih.gov/compound/Adenosine [10]

**Figure 2:**
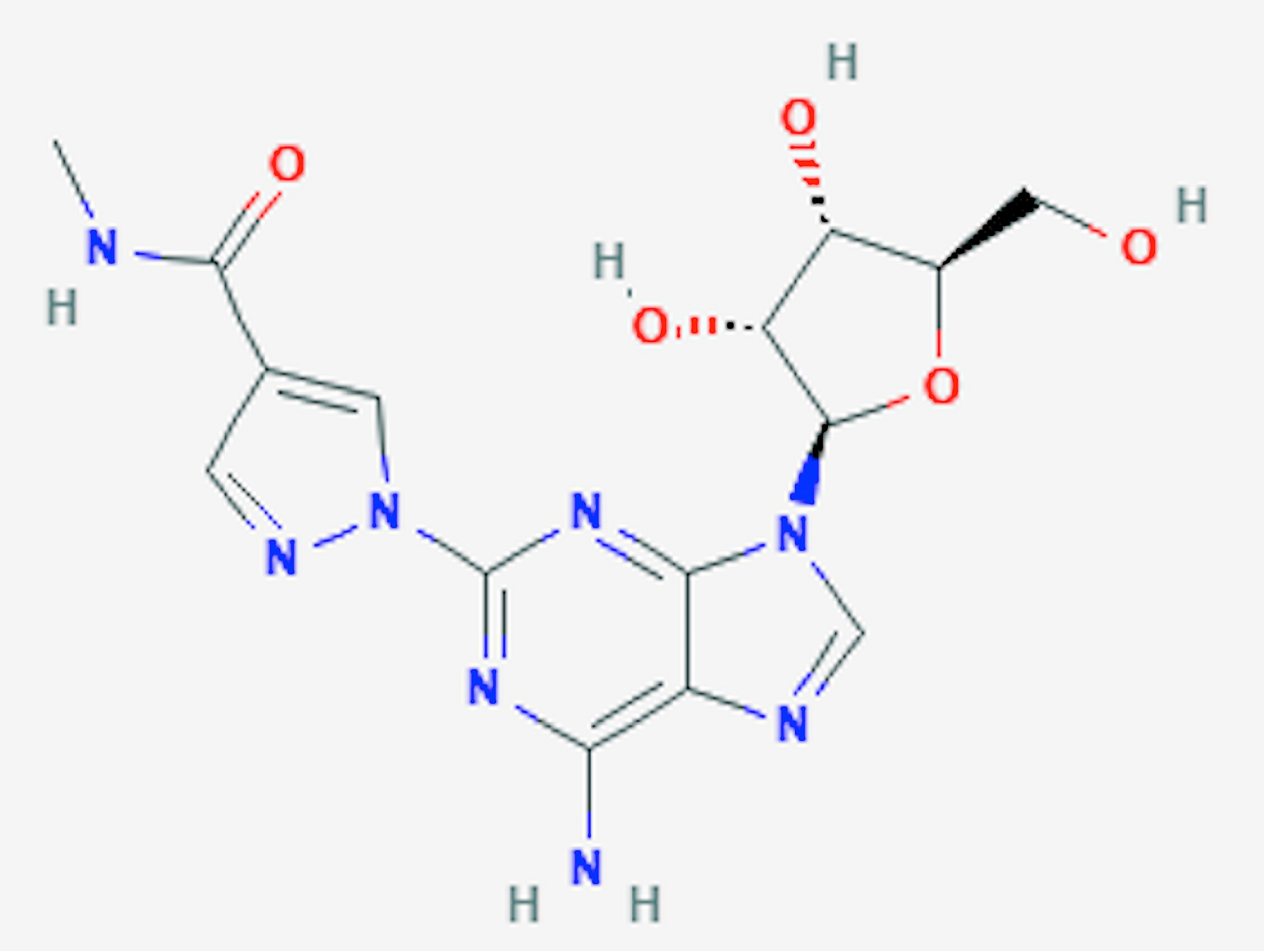
The chemical structure of regadenoson National Center for Biotechnology Information (2020). PubChem Compound Summary for CID 219024, Regadenoson. Retrieved October 13, 2020, from https://pubchem.ncbi.nlm.nih.gov/compound/Regadenoson [10]

Regadenoson is a 2-[N-1-4(4-N-methyl carboxamide pyrazolyl)] adenosine derivative that causes the dilatation of coronaries by binding to A2a receptors situated on the smooth muscle cells of the coronary arteries [9]. As shown in Figures 1-2, the N-pyrazole class is substituted for C-pyrazole, which confers more affinity for the A2A receptors [10-11]. Regadenoson was approved by FDA in April 2008, largely due to its safety profile. As mentioned previously, unlike regadenoson, older agents, such as adenosine, dipyridamole, and dobutamine, have undesirable side effects such as chest pain, bronchospasm, atrioventricular (AV) block, hypotension, and arrhythmias [11]. Regandonson exerts its effects by binding to A2a receptors; these receptors are stimulatory guanine nucleotide-binding proteins (G proteins), which upon binding to regadenoson, activates adenylyl cyclase, thereby increasing cyclic adenosine 5T monophosphate (CAMP), leading to the phosphorylation of protein kinase A (PKA) and the production of membrane hyperpolarization [12-13]. Therefore, the activation of A2a receptors dilates the coronary arteries, causing hyperemia and increased coronary blood flow (CBF). The increased CBF occurs in a dose-dependent manner, with maximal dilatation seen when only 25% of the receptors are bound by regadenoson [14-15]. In comparison with adenosine, as shown in Table 1, regadenoson is more potent and needs lesser molecules to achieve 50% of the maximum vasodilator effect (5.9 nM of adenosine vs 6.4 nM of regadenoson) [16-17]. It is reported that the onset of action of coronary vasodilation for regadenoson is approximately 30 seconds [18-19]. More specifically, the relationship between time and the plasma concentration of regadenoson is reflected by a three-compartment model. The initial phase of coronary vasodilation lasts approximately three minutes and indicates the onset of regadenoson response; the middle phase is denoted by a loss of effect of coronary vasodilation, which lasts for approximately 30 min. Simultaneously, the final phase occurs with a decrease in plasma regadenoson concentration [19]. Renal excretion is responsible for 58% of the total elimination of regadenoson with a clearance rate of 38l/h [19].

**Table 1:** Comparison between regadenoson and adenosine Source: [10] AV: Atrioventricular; HR: Heart rate *Duration of action is defined as the duration of circulatory flow maintained >2.5 times of the baseline

Parameters	Regadenoson	Adenosine
Potency	10 times more potent than adenosine	Less potent
Receptor selectivity	Selective A_2a_ adenosine receptor agonist	Nonselective agonist
Dose	400 ug as a single dose	140 ug/kg/min
Duration of infusion	10 seconds bolus	4 – 6 min
Radiotracer injection	30s after bolus	Third minute of infusion
Time to peak plasma concentration (T_max _)	33s	30s
Metabolism	No hepatic metabolism	Deaminated to inosine by adenosine deaminase
Excretion	57% appears unchanged in urine	Cellular uptake
Half-life	1^st^ phase: 2 – 4 min 2^nd^ phase: 30 min 3^rd^ phase: 2 h	< 10 s
Duration of action	2.3 min*	6 s*
Coronary blood flow	Increased to >2 times baseline in 30 s and decreased to < 2 times baseline in 10 min	Maximum response at 2 – 3 min after infusion onset; return to baseline within 1 – 2 min following cessation of infusion
Coronary hyperemia	2 – 5 min longer than adenosine	Lesser effect
Heart rate	Faster and greater peak increase in HR	Less fast and lesser peak in HR
Adverse effects	Less hypotension, AV blocks, bronchoconstriction	hypotension, AV blocks, bronchoconstriction

Indications for regadenoson testing 

Regandonson is usually used as a pharmacological stress agent (PSA) coupled with radionuclide imaging or non-radionuclide imaging. Real-time myocardial contrast echocardiography (RTCME) utilizes regadenoson without a radionuclide, thereby eliminating the need for radiation exposure. This can rapidly diagnose coronary stenoses while maintaining high specificity and sensitivity [20]. It is also used as PSA in myocardial perfusion imaging (MPI) studies especially in post-cardiac transplantation, as it demonstrated equal efficacy compared with older agents and a favorable side effect profile, including fewer arrhythmias [21]. Despite its dosing as a single fixed-dose bolus, it is an excellent PSA in PET imaging, as it results in a significant increase in myocardial blood flow, which is independent of the patient’s distribution volume, which is comparable to the effects seen with dipyridamole [22]. Indications of regadenoson include the following: to risk-stratify patients with presumed coronary artery disease with an inability to complete adequate exercise due to musculoskeletal, pulmonary, mental, pulmonary disorders, and lack of motivation to exercise. Additionally, it’s indicated to risk stratify patients that are clinically stable post acute myocardial infarction, to risk-stratify patients with presumed coronary artery disease with baseline EKG anomalies such as ventricular pre-excitation syndromes (Wolff- Parkinson-White syndrome), left bundle branch blocks, and the presence of permanent pacemakers. Finally, to risk stratify patients presenting to the emergency room with presumed acute coronary syndrome following exclusion by serial cardiac enzymes, EKGs, clinical history, and examination [23].

Interpretation of results

There are three main different ways to assess the perfusion defects when interpreting regadenoson radionuclide imaging stress testing: qualitative analysis, semi-quantitative analysis, or quantitative analysis. Usually, the methods of quantitative analysis serve as complements to assist in qualitative or semi qualitative visual analysis.

**Figure 3:**
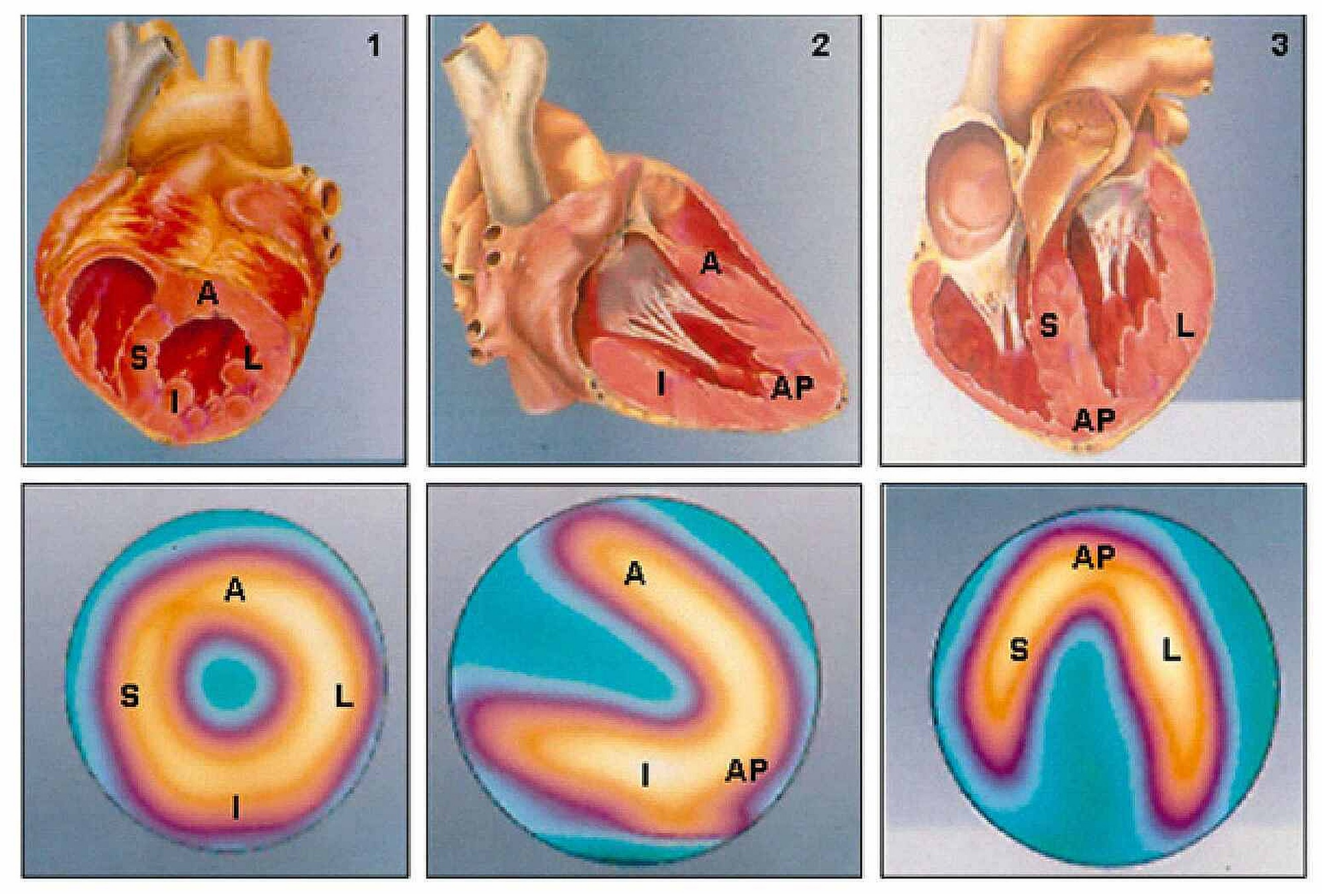
Two-dimensional reconstructive images of scintigraphy (lower images) and its correspondence to three-dimensional heart model (upper images) Two-dimensional reconstruction of scintigraphy images representing normal perfusion patterns (lower images), in line with the minor axis (1), vertical long axis (2), and horizontal long axis (3) cross-sections and their respective corresponding anatomical cross-sections (upper images) A: anterior; AP: apical; I: inferior; L: lateral; S: septal Adapted from Mastrocola LE [24]

1. Visual or qualitative analysis: By simply inspecting images resulting from perfusion tomography and ventricular function exams. Characterization of the uptake of radiopharmaceutical material during the resting and stress stages is shown in Figure 3. The interpretation of the visual analysis is shown in Table 2. 

**Table 2:** The interpretation of radionuclide stress testing by visual analysis * Hibernating myocardium also shows persistently reduced uptake. Assessment of viable myocardium with thallium-201 can help to distinguish it from fibrotic tissue, which sometimes necessitates adding another phase or stage, namely, that of late redistribution or re-injection, interpreted in the same manner [23].

Visual findings	Interpretation
No significant change during both stress and resting/distribution phases	Normal myocardium
Relative uptake in stress images and normal uptake during resting/redistribution phase	Ischemic myocardium
Reduced relative uptake during both stress and resting/distribution phases*	Fibrotic myocardium
Reduced relative uptake will be observed during the stress phase, with partial improvement during the resting/redistribution phase	Fibrotic tissue coexists with an ischemic yet viable myocardium.

**Figure 4:**
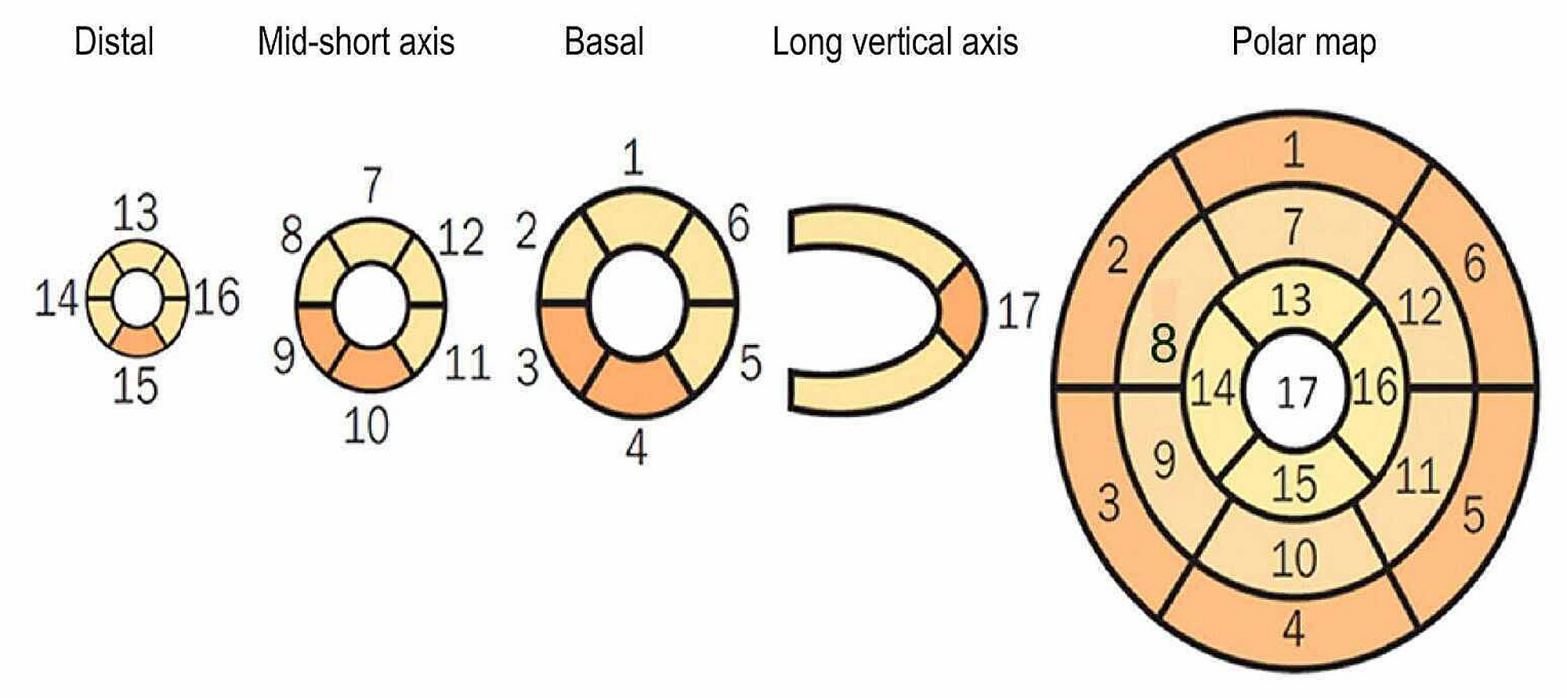
Numerical segmentation model of the left ventricular myocardium in 17 segments Considering the tomographic slices of the minor and long vertical axes (distal or apical, middle, and basal or proximal portions), representing the myocardial regions. Furthermore, correspondence of segments may be seen as presented in the polar map, which represents radiopharmaceutical distribution throughout the left ventricular myocardium in the form of a polar map, whose center corresponds to the apex and whose peripheries correspond to the basal portions [24].

2. Semiquantitative analysis: The aim of this method is to numerically assess the intensity of radiopharmaceutical uptake (perfusion) using the established standards 17-segment model as shown in Figure 4. Specific scores have been developed: 0 = normal; 1 = mildly reduced radiopharmaceutical uptake; 2 = moderately reduced uptake; 3 = severely reduced uptake; 4 = absence of radiopharmaceutical uptake. Further calculations are achieved by the sum of values attributed to each segment: the sum of the values attributed to each segment during the stress phase is known as the “summed stress score” (SSS); this is repeated during the baseline or redistribution phase to obtain the “summed rest/redistribution score” (SRS). The interpretation of SSS is shown in Table 3.

**Table 3:** The interpretation of radionuclide stress testing by semi-quantitative analysis * SSS values < 4 are understood as normal. However, it might not be zero and that is because there are myocardial regions that show lower radiopharmaceutical concentrations and, consequently, receive values other than zero [25-26]. SSS: Summed stress score

Summed stress score (SSS)	Interpretation
< 4	Normal*
4-8	Mildly abnormal
9-13	Moderately abnormal
> 13	Severely abnormal

 3. Quantitative analysis: This method uses polar maps that are two- or three-dimensional reconstruction of the left ventricle (LV). Radiopharmaceutical uptake, which is indicative of perfusion is shown on a color scale. Special programs that are capable of reforming those images to allow for the quantification of areas with reduced uptake. It is quantified by comparing the images to the database of normal individuals of the same age and sex. In addition, perfusion defects are quantified by the number of pixels in a specific region and by calculating standard deviations in relation to normal perfusion areas [25-26].

Contraindications

Regadenoson has a large safety profile, however, its use is absolutely contraindicated in second-degree AV block, third-degree AV block, and sinus node disease without a functioning pacemaker, patients with a bronchospastic disease with active wheezing, hypotension (systolic blood pressure less than 90 mm Hg), hypertension that is uncontrolled (systolic blood pressure greater than 200 mmHg or diastolic blood pressure greater than 110 mmHg), recent use-within 48 hours of dipyridamole or dipyridamole-containing drugs such as Aggrenox, acute coronary syndrome, unstable angina, and within four days of acute myocardial infarction [23]. Relative contraindications for regadenoson stress test are severe sinus bradycardia (heart rate less than 40beats/min, Mobitz type 1 2nd degree AV block, ingestion of caffeine-containing drinks or foods within 12 hours, history of seizure disorder (regadenoson might lower seizure threshold), and severe aortic stenosis [23].

Characteristics of regadenoson in specific patients population

Obesity

One report showed the response to regadenoson was less pronounced in diabetics and obese patients [27]. However, another randomized control trial of 356 patients (n=365) showed that there were no significant changes in systolic blood pressures (SBP) or heart rates (HR) across the categories of different body mass index (BMI) [28]. Based on the aforementioned reports, the effects of regadenoson on obese patients has not yet been established.

Reactive Airway Disease

The initial findings by Thomas et al. and Leaker et al., in the RegCOPD (Regadenoson in Chronic Obstructive Lung Disease patients) trial and the RegAsthma (Regadenoson in Asthma patients) trial, respectively, demonstrated the safety and efficiency of regadenoson in patients with reactive airway disease. Furthermore, those findings were validated by Prenner et al. in the subsequent report as shown in Table 4.

**Table 4:** Relevant clinical trials studying the effects of regadenoson on reactive airway diseases FEV1: Forced expiratory volume 1; COPD: Chronic obstructive pulmonary disease

Authors	Design	Sample size	Primary outcome	Results
Thomas et al. [29]	Randomized, double-blinded placebo-controlled crossover trial	49 COPD Patients	Decline in FEV1	No difference between both groups
Leaker et al. [30]	Randomized, double-blinded placebo-controlled crossover trial	48 Asthma Patients	Decline in FEV1	No difference between both groups
Prenner et al. [31]	Randomized, double-blind placebo-controlled study	576 Asthma Patients	Decline in FEV1	No difference between both groups
Prenner et al. [31]	Randomized, double-blind placebo-controlled study	467 COPD Patients	Decline in FEV1	No difference between both groups

Based on the above-mentioned reports, it is concluded that the use of regadenoson is safe for use in patients with reactive airway diseases irrelevant of the severity of the condition.

Renal Impairment

Gleaned from the reports listed in Table 5, it’s reasonable to conclude that the use of regadenoson is safe and effective in patients with renal impairment. Ultimately, in a label update in January 2017, the U.S. FDA (USFDA) has outlined that there is no need for dose adjustments when using regadenoson in patients with renal impairment and/or those on dialysis [35].

**Table 5:** Relevant clinical reports studying effects of regadenoson in patients with renal impairment ESRD: End-stage renal disease; CKD: Chronic kidney disease; HR: Heart rate; SBP: Systolic blood pressure; GFR: Glomerular filtration rate * No serious adverse events or deaths were reported 24 hours post-dose

Authors	Design	Sample size	Primary outcomes	Results
Aljaroudi et al. [32]	Observational, retrospective study.	277 patients with ESRD, 134 patients with normal kidney function	Change in HR change in SBP	No significant difference
Doukky et al. [33]	Observational, retrospective study	146 ESRD patients, 97 patients GFR ≥ 30	Regandonson adverse effects ST-segment deviation, arrhythmias, AV block, hypotension	No significant difference
Ananthasubramaniam et al. [34]	Randomized, double-blinded, placebo-controlled study.	432 subjects with stage 3 CKD (regadenoson n = 287; placebo n = 145), and 72 subjects with stage 4 CKD (regadenoson n = 47; placebo n = 25)	Regandonson adverse events (24 hours post-dose)	The incidence of adverse events was higher in the regadenoson group compared to the placebo

Caffeine Use

The results obtained from the reports listed in Table 6 indicate that the use of caffeine is safe for patients undergoing regadenoson stress testing. The use of caffeine was further studied as an alternative to aminophylline in a reversal of the side effects of regadenoson. Doran et al. randomized 241 patients to receive either 100 mg of intravenous (IV) aminophylline, 60 mg of IV caffeine, or a caffeinated beverage for reversal of regadenoson-associated symptoms. It was observed that IV caffeine and IV aminophylline had similar effects on the complete resolution (CR) of symptoms for regadenoson. However, oral caffeine was inferior to IV aminophylline for CR [38].

**Table 6:** Relevant clinical reports studying effects of caffeine on regadenoson use CMR: Cardiac magnetic resonance; BP: Blood pressure

Authors	Methods	Sample size	Primary outcome	Results
Posch et al. [36]	Observational prospective study; patients presenting for regadenoson stress myocardial perfusion imaging were asked their amounts of daily caffeine intake.	101 patients; 89% reported caffeine intake, with 13% reporting heavy caffeine intake (> 400 mg daily)	Chest pain, aminophylline administration, resting/peak heart rate, diastolic BP response	Less chest pain, aminophylline administration, lower resting and peak heart, and lower diastolic BP in caffeine users
Dijk et al. [37]	98 consecutive patients with suspected coronary artery disease referred for either adenosine or regadenoson perfusion CMR were included in this analysis.	33 patients undergoing regadenoson CMR (9 patients with coffee consumptions < 4h, 24 patients with no coffee consumption	T1 reactivity; subtracting T1rest from T1stress	No significant difference in patients who reported no coffee intake compared to patients with less than 4 hours since their last coffee intake

Seizures

Although it has not been studied extensively, Page et al. reported a case series of three patients who developed seizures after administration of regadenoson. It was identified that regadenoson proposed a potential risk for developing seizures [39]. Based on animal models, the mechanism is proposed to be that activating A2A receptors will enhance glutamatergic excitotoxicity in addition to inhibiting the neuroprotective effect of A1 receptors, thus exacerbating seizures [40]. Nevertheless, due to the lack of evidence, the use of regadenoson is yet not contraindicated in patients with a seizure disorder.

Low Socioeconomic Population

To the best of our knowledge, there were no reports that studied the effects and safety profile of regadenoson on low socioeconomic populations. Low socioeconomic status is considered a significant risk factor for CAD as reported by Djekic et al. [41]. Furthermore, Mols et al. studied social factors (grade of education, employment, and cohabiting status) of 568 normal healthy subjects and coronary artery calcification (CAC). The study concluded an association between social factors with the prevalence and severity of CAC in asymptomatic middle-aged individuals [42]. Those reports highlight the importance of socioeconomic factors as an independent risk factor for CAD. A cardiovascular stress test costs about $3,800 on average nationally, according to NewChoiceHealth.com but can get as high as $10,900. Prices range based on geographical location, with urban, metropolitan areas charging higher prices than rural areas [43]. Thus, the need for further studies to determine the efficiency, safety, and cost benefits of regadenoson in this specific patient population is warranted.

Other Agents

Apadenoson (APA) is a highly selective A2a receptor stimulator medication. Consequently, this drug holds a potential utility for pharmacologic stress myocardial perfusion imaging. Similarly, binodenoson (BND) is a drug that selectively targets the adenosine 2A receptors. When comparing the properties of the three drugs, APA, BND, and regadenoson, we can observe that APA has a higher selectivity for the A2A receptor while BND is slightly less selective and regadenoson is the least selective [44-47]. Additionally, when assessing the affinity of each of these drugs to the A2A receptor, it is concluded that both APA and BND have equal affinity for the receptor while regadenoson has the least affinity for the receptor [44-47]. The potency of each drug is very much similar to the selectivity of the drug for the A2A receptor. Regadenoson has the least potency while APA possesses the most potency. The key difference between these three drugs lies in the onset and duration of action. The quickest onset of action is seen with regadenoson at 33 seconds while both APA and BND have an average onset of one to two minutes [44-47]. In regards to the duration of action, regadenoson is the shortest at 2.3 minutes while BND comes in a second with an average duration of less than five minutes with APA having the longest duration of action at approximately 10 to 20 minutes [44-47]. When comparing the route of administration all three of the drugs are given as an intravenous bolus. However, the dose is calculated depending on the weight for both BND and APA, regadenoson is a fixed-dose drug that requires no weight-based calculation [44-47].

## Conclusions

The regadenoson stress test is the most used pharmacological stress test. It works by vasodilating the coronary arteries by stimulating the A2A receptors, increasing HR, and decreasing blood pressure. Through our literature review, we were able to analyze several patient characteristics that may or may not have an effect in interpreting the results of this pharmacological stress test. Regadenoson’s safety has been documented in patients with asthma or chronic obstructive pulmonary disease (COPD) as well as patients with chronic kidney disease (CKD) or end-stage renal disease (ESRD). Moreover, it has been established that the concomitant use of caffeine with regadenoson is tolerable. However, the effects of regadenoson on obese patients still require further attention due to contradictory findings in the current literature. Nevertheless, the safety of regadenoson in patients with pre-existing seizures warrants additional studying, as the data remain limited. Lastly, to the best of our knowledge, there are no reports available that analyzed the cost benefits and safety profile of regadenoson in patients with low socioeconomic status. Low socioeconomic status can be considered an independent risk factor for CAD, which merits attention to study the efficiency, safety, and cost benefits of regadenoson in this specific patient population. It is crucial to carefully examine the patient’s profile to determine the most appropriate type of stress test and to evaluate the safety profile of using the regadenoson pharmacological stress test in certain groups of patients. Amongst apadenoson and binodenoson, regadenoson might have the least selectivity and affinity to A2A receptors. However, its short duration of action and absence of the need for weight adjustments highlight its convenience to use over the other agents.
